# Is there evidence of anti-malarial multidrug resistance in Burkina Faso?

**DOI:** 10.1186/s12936-021-03845-5

**Published:** 2021-07-19

**Authors:** Charlotte Rasmussen, Pascal Ringwald

**Affiliations:** Global Malaria Programme, World Health Organization, Geneva, Switzerland

**Keywords:** Malaria drug resistance, Anti-malarial efficacy, Therapeutic efficacy studies, Anti-malarial treatment failures

## Abstract

Recently, Gansané and colleagues published an article on inadequate efficacy of two different forms of artemisinin-based combination therapy (ACT) in Burkina Faso. The development of *Plasmodium falciparum* resistance to different ACT partner drugs at levels that could affect the efficacy of two ACT would both be startling and a cause for great concern. In reviewing the available data collected since 2008 on ACT efficacy in Burkina Faso, the analysis shows that the reported efficacy of the tested ACT varies greatly. Most of the studies have considerable methodological deviations and challenges, especially in PCR correction done to distinguish between recrudescence and re-infection, and in the failure to omit re-infections in the calculation of efficacy rates. So far, there is no convincing evidence in the articles reviewed that multidrug resistance has emerged in Burkina Faso. However, the potential consequence of failing ACT means that the results published by Gansané et al. urgently need to be confirmed. Furthermore, articles reporting on efficacy data need to include an examination of the potential consequences of any methodological deviations.

## Background

Anti-malarial drug resistance is a major challenge for malaria control and elimination. Monitoring drug efficacy is important to detect resistance and adapt national treatment policy. The World Health Organization (WHO) has developed a standard protocol for therapeutic efficacy studies (TES) to facilitate the collection of comparable, high quality data. It is recommended to change the treatment policy if good quality studies detect an efficacy below 90 % for the first-line treatment [1].

The WHO recommends artemisinin-based combination therapy (ACT) for the treatment of uncomplicated *Plasmodium falciparum*. In the Greater Mekong Sub-region, artemisinin partial resistance helped facilitate the selection and spread of resistance of ACT partner drugs, the major factor of ACT failures [1]. Artemisinin partial resistance has recently been confirmed in Rwanda with the independent emergence and spread of a *Pfkelch13* R561H mutant. While this appears to have affected the clearance rate, so far it did not affect the efficacy of artemether-lumefantrine (AL) or dihydroartemisinin-piperaquine (DP) [2]. Assessing the efficacy of partner drugs is as important as investigating emergence and spread of artemisinin partial resistance.

Recently, Gansané et al. published an article on inadequate efficacy of AL and DP in Burkina Faso [3]. Finding simultaneous high failure rates of two ACT whose partner drugs belong to different chemical families in at least two sites is noteworthy (74.4–91.6 % for AL on day 28 and 83.6–97.2 % for DP on day 42 using Kaplan-Meier analysis). If confirmed, the presence of resistance to both lumefantrine and piperaquine at a prevalence that could cause such high failure rates is alarming.

To assess evidence of ACT efficacy in Burkina Faso, published results and methodologies for TES conducted since 2008 were analysed. This information was extracted from the WHO anti-malarial drug efficacy database publicly available through the WHO malaria threat maps (https://apps.who.int/malaria/maps/threats).

## Current evidence of ACT efficacy in Burkina Faso

In addition to data presented by Gansané et al. [3], 15 published articles with efficacy data for five recommended artemisinin-based combinations used alone or in combination with methylene blue or primaquine were identified and reviewed [4–18]. An overview of the data presented in the 15 articles can be found in Table 1. The Table lists the deviations from the standard WHO TES protocol and an assessment of the potential impact that these deviations may have had on the proportion of the study patients recorded to have an adequate clinical and parasitological response (ACPR) to treatment. Where possible, the data are presented in the Table by sites rather than combined across sites. For one multi-country study, the reported data does not allow for an analysis of the efficacy data collected in Burkina Faso only [17]. For another multi-country study, the efficacy data are only available as a combined reported efficacy estimate across sites in different countries and drugs [18].

**Table 1:** Deviation to WHO protocol and impact on treatment outcome

Study setting (Ref)	Study design (follow-up)	Study pop. (n)	ACT	Reported % ACPR	Deviation from WHO protocol	Impact on ACPR
Nouna health centre Aug–Oct 2011 [4]	RCT (28 days)	6–59 months (193)	ASAQ vs ASAQ + MB	85.1 *vs* 71.4	mITT analysis, ETF misclassification	Decrease
					PCR correction with *msp1* and *msp2*	Decrease
					Re-infection classified as ACPR	Increase
Nouna hospital. OctNov 2016 [5]	RCT (28 days)	6–59 months (100)	ASAQ + PQ vs ASAQ + MB	95.9 *vs* 95.7	ITT analysis	Decrease
					PCR correction with *msp1* and *msp2*	Decrease
					Re-infection classified as ACPR	Increase
Tiéfora and Mangodara health centres, Comoé Province. Sep–Dec 2009 [6]	Single arm (28 days)	6–59 months (105)	AL	90.5	PCR correction with *msp2* only	Decrease
					Re-infection classified as ACPR	Increase
Tiéfora and Mangodara health centres, Comoé Province. Sep–Dec 2012 [7]	Single arm (28 days)	6–59 months (105)	AL	86.7	PCR correction with *msp2* only	Decrease
					Re-infection classified as ACPR	Increase
Ouagadougou and Nanoro. 2012–2014 [8]	Multicentre, multicounty, single arm (28 and 42 days)	> 28 days and <5 kg (20)	AL	100 day 28-100 day 42^a^	Re-infection classified as ACPR	Increase
Colsama and Sakary health centres, Bobo-Dioulasso Jun–Dec 2016 [9]	RCT (28 days)	> 6 months (281)	AL *vs* ASAQ	85.2 *vs* 97.0	No PCR correction	Decrease
Nanoro health district Sep 2008-Jan 2010 [10]	RCT (28 and 42 days)	6–59 months (340)	AL *vs* ASAQ	89.8 *vs* 89.7 - day 28 66.7 *vs* 63.0 day 42	PCR correction with msp1 and msp2	Decrease
					Re-infection classified as ACPR	Increase
					Unsupervised treatment	Decease
Nanoro health district Sep 2010-Oct 2012 [11–13]	RCT (28 days)	> 6 months (680)	AL *vs* ASAQ	77.8 *vs* 84.1	PCR correction with *msp1* and *msp2*	Decrease
					Re-infection classified as ACPR	Increase
					Unsupervised treatment	Decease
Dafra medical centre, Bobo-Dioulasso Dec 2008-Dec 2010 [14]	RCT (42 days)	6 months–15 years (440)	AL *vs* ASAQ	91.1 *vs* 98.1	Longer follow-up	Decrease
					Re-infection classified as ACPR	Increase
Nanoro health district Jun 2010-Aug 2013 [15]	Multicentre, multicounty, RCT (63 days)	Pregnant women (802)	AL *vs* ASAQ *vs* ASMQ	93.2 *vs* 96.7 *vs* 92.5	Longer follow-up	Decrease
					Re-infection classified as ACPR	Increase
Balinghi. Oct 2010–Oct 2013 [6]	Multicentre, multicounty, RCT (63 days)	6–59 months (245)	AL *vs* ASMQ	95.9 *vs* 97.6	Danger sign classified ETF at day 1	Decrease
					Longer follow-up	Decrease
					Re-infection classified as ACPR	Increase
Banfora Oct 2010–Oct 2013 [16]	Multicentre, multicounty, RCT (63 days)	6–59 months (115)	AL *vs* ASMQ	94.9 *vs* 91.2	Danger sign classified ETF at day 1	Decrease
					Longer follow-up	Decrease
					Re-infection classified as ACPR	Increase
Ouagadougou and Nanoro Nov 2013-Jun 2015 [17]	Multicentre, multicounty, RCT (42 days)	6–12 months (90)	DP different formulation	98:3 *vs* 100^b^	Re-infection classified as ACPR	Increase
Bobo Dioulasso and Banfora-Niangoloko Oct 2011–Feb 2016 [18]	Multicentre, multicounty, RCT (28 and 42 days)	> 6 months (1507)	AL *vs* ASAQ *vs* ASPY *vs* DP	> 99.5 day 28 98.6 day 42^c^	Re-infection classified ACPR if failure recorded on last day of follow-up	Increase

*RCT* randomized controlled trial, *ACT* artemisinin-based combination therapy, *AL* artemether-lumefantrine, *ASAQ* artesunate-amodiaquine, *ASPY* artesunate-pyronaridine, *ASMQ* artesunate-mefloquine, *DP* dihydroartemisinin-piperaquine, *ACPR* adequate clinical and parasitological response, *mITT* modified intent-to-treat, *ITT* intent-to-treat, *ETF* early treatment failure, *PCR* polymerase chain reaction, *msp* merozoite surface protein, *P. Plasmodium*

^a^Data from all sites in Burkina Faso and Benin

^b^Data from all sites in Burkina Faso, The Gambia, Democratic Republic of Congo, Mozambique, Tanzania

^c^Data from all sites in Burkina Faso, Guinea and Mali and for all ACT

Overall, the most frequent study deviations observed were the classification of re-infection as ACPR instead of excluding them from the analysis; this could lead to a significant overestimation of the efficacy in studies with high number of re-infections. All the other deviations identified could lead to an underestimation of the efficacy. These include using a PCR correction methodology different to the one currently recommended by the WHO, which requires the use of three markers: *msp1*, *msp2* and *glurp*. Using fewer markers could lead to a lower estimated efficacy as a recrudescence requires that at least one allele on every locus is common in the parasites on day 0 and the day of failure.

The recommended first-line treatments for uncomplicated *P. falciparum* in Burkina Faso are artesunate-amodiaquine (ASAQ), AL or DP. In the articles reviewed, the reported efficacy for ASAQ alone or in combination with primaquine or methylene blue ranges from 63.0 to 98.1 %. One of the studies finding lower than 90 % efficacy for ASAQ is a randomized trial in Nouna in 2011. This study assessed ASAQ alone or with methylene blue and reported efficacies of 85.1 and 71.4 %, respectively [4]. The endpoints in the study were analysed using an intent-to-treat methodology classifying patients vomiting, refusing a second dose, or violating the protocol as early treatment failure. A similar study conducted at the same site in 2016 with ASAQ with primaquine or methylene blue did not find the same high early or late treatment failure rate, reporting a failure rate of less than 5.0 % [5].

The reported efficacy for AL ranges from 66.7 to 100 %. Two studies finding less than 90 % efficacy for both ASAQ and AL were done in Nanoro health district in 2008–2010 and 2010–2012. The studies reported failure rates on day 28 of 10.2–22.2 % for AL and 10.3–15.9 % for ASAQ. These trials were effectiveness trials for which treatment is unsupervised (meaning only the first of 6 doses of AL and the first of 3 doses of ASAQ were supervised by health staff, contrarily to efficacy trials for which all doses are supervised) [10, 11]. Therefore, higher treatment failure rates were expected.

A study performed in Bobo-Dioulasso in 2016 also compared ASAQ and AL, finding high failure rates in the AL arm but not in the ASAQ arm [9]. As no PCR correction was undertaken it is likely that some reported failures were due to re-infection. The difference of efficacy between ASAQ and AL could be due to the shorter elimination half-life of lumefantrine leading to more re-infections in areas of high transmission.

One of two studies done in Comoé Province found less than 90 % efficacy for AL. The studies were done before and after a large community-based distribution of AL, and showed a statistically non-significant decrease of AL efficacy after the distribution in 2012 (86.7 %, n = 105) compared to before in 2009 (90.5 %, n = 105). The studies in Comoé used only *msp2* in the PCR correction [6, 7].

In addition to the AL and ASAQ studies described above finding low efficacy, a number of studies found higher than 90 % efficacy; however, also in those studies deviations mean that the data need to be interpreted with caution. None of the previous studies with DP or any of the studies with artesunate-mefloquine (ASMQ) or artesunate-pyronaridine (ASPY) found an efficacy lower than 90 %.

The recent article published by Gansané et al. [3] reports on studies done in 2017–2018 in three sites (Niangoloko, Nanoro, Gourcy), finding PCR-corrected treatment efficacy of 74.4–91.6 % for AL on day 28, and 83.6–97.2 % for DP on day 42 using Kaplan-Meier analysis. Some aspects of the studies and results are worth noting. *Pfplasmepsin2* is a validated marker of piperaquine resistance in the Greater Mekong sub-region, but no correlation was reported between DP failure and *Pfplasmepsin2* in the study. The study did not include day 7 lumefantrine blood levels, which in the absence of a validated marker for lumefantrine resistance would be useful to help confirm resistance as cause of AL failures. A 12 % discordance between positive and negative results reported during quality control of the microscopy conducted at the Noguchi Memorial Institute for Medical Research in Ghana is concerning. No further indications were provided in the article on the quality of staining, such as the presence of debris on slides or stain used, which could further increase the percentage of discordance on parasite counting. The WHO recommends that if more than 10 % of a sub-sample has non-concordant outcomes, all study results should be reviewed [19]. DNA detected by PCR on day of failure is not enough to confirm the presence of asexual parasites, as DNA is detectable in blood for a long period of time [20]. Presence of gametocytes can lead to misclassification of a failure as recrudescence. Consequently, gametocytaemia must be detected in case of a high treatment failure rate through more extensive reading of slides taken at day of failure against 1,000 white blood cells (WBC) or 100 high-power fields [21, 22]. The absence of amplification of *glurp* reported in the article at such high level is very rarely observed in other studies (47.6 % amplified). The data presented in the article do not differentiate between *glurp* amplification failed and not attempted; actually, *glurp* amplifications were not attempted for 168 paired samples (Institute Pasteur Paris, pers. comm.). Therefore, the calculated *glurp* amplification success rate should be closer to 92.3 % for 118 paired samples tested, and less than 35 % of the failures were corrected with the 3 recommended markers. Failure to amplify the 3 markers particularly at day of failure should also trigger quality control of microscopy and confirmation of presence of parasites.

## Need for further studies

The analysis showed inconsistent failure rates with AL and ASAQ and that almost all studies in Burkina Faso had methodological deviations. So far, there is no convincing evidence in the articles reviewed that multidrug resistance has emerged in Burkina Faso, in particular amodiaquine, lumefantrine and piperaquine resistance. The most recent results published by Gansamé et al. [3] urgently need to be confirmed by studies with the recommended monitoring and quality control of slide reading.

The WHO recently released a report highlighting major challenges detected during TES, which can have implications for study outcomes [1]. Quality and quality control of slide reading remain a main challenge in many studies. Using a standard protocol does improve comparability over time and among sites. Conducting quality control monitoring before starting the study, at mid-term and at the end of the study does improve the quality of data. PCR correction of treatment failure to distinguish between re-infection from recrudescence is mandatory during efficacy studies. Effectiveness studies are closer to ‘real-life’ situations but are not relevant to assess ACT efficacy or anti-malarial drug resistance. Reports on efficacy studies with unexpectedly high treatment failure rates need to include an examination and explanation of the type of failures recorded, in particular the criteria on which early treatment failures were classified. Impact of any potential deviation to standard methodologies should be examined.

## Data Availability

All data analysed during this study are included in this published article.

## Abbreviations

ACPR: Adequate clinical and parasitological response
ACT: Artemisinin-based combination therapy
AL: Artemether-lumefantrine
ASAQ: Artesunate-amodiaquine
ASMQ: Artesunate-mefloquine
ASPY: Artesunate-pyronaridine
DP: Dihydroartemisinin-piperaquine
PCR: Polymerase chain reaction
TES: Therapeutic efficacy studies
WBC: White blood cells
WHO: World Health Organization

## References

[1] WHO. Report on antimalarial drug efficacy, resistance and response: 10 years of surveillance (2010–2019). Geneva, World Health Organization. 2020. https://www.who.int/publications/i/item/9789240012813.

[2] Uwimana A, Legrand E, Stokes BH, Ndikumana JM, Warsame M, Umulisa N (2020). Emergence and clonal expansion of in vitro artemisinin-resistant *Plasmodium falciparum* kelch13 R561H mutant parasites in Rwanda. Nat Med.

[3] Gansané A, Moriarty LF, Ménard D, Yerbanga I, Ouedraogo E, Sondo P (2021). Anti–malarial efficacy and resistance monitoring of artemether–lumefantrine and dihydroartemisinin–piperaquine shows inadequate efficacy in children in Burkina Faso, 2017–2018. Malar J.

[4] Coulibaly B, Pritsch M, Bountogo M, Meissner PE, Nebié E, Klose C (2015). Efficacy and safety of triple combination therapy with artesunate-amodiaquine-methylene blue for falciparum malaria in children: a randomized controlled trial in Burkina Faso. J Infect Dis.

[5] Mendes Jorge M, Ouermi L, Meissner P, Compaoré G, Coulibaly B, Nebie E (2019). Safety and efficacy of artesunate-amodiaquine combined with either methylene blue or primaquine in children with falciparum malaria in Burkina Faso: a randomized controlled trial. PLoS ONE.

[6] Siribié M, Diarra A, Tiono A, Soulama I, Sirima S (2012). Efficacité de l’artéméther-luméfantrine dans le traitement du paludisme simple de l’enfant en milieu rural au Burkina Faso en 2009. Bull Soc Path Exot.

[7] Siribié M, Diarra A, Tiono AB, Soulama I, Sirima SB (2015). Effet d’une distribution communautaire à large échelle de l’artéméther-luméfantrine sur son efficacité thérapeutique chez les enfants vivant en milieu rural au Burkina Faso. Bull Soc Pathol Exot.

[8] Tiono AB, Tinto H, Alao MJ, Meremikwu M, Tshefu A, Ogutu B (2015). Increased systemic exposures of artemether and dihydroartemisinin in infants under 5 kg with uncomplicated *Plasmodium falciparum* malaria treated with artemether-lumefantrine (Coartem^®^). Malar J.

[9] Zongo I, Compaoré YD, Nikiéma F, Zongo M, Barry N, Somé FA (2020). Efficacy of artemether-lumefantrine and artesunate-amodiaquine as first line therapy of uncomplicated malaria in Burkina Faso, 11 years after policy change. Pan Afr Med J.

[10] Tinto H, Diallo S, Zongo I, Guiraud I, Valea I, Kazienga A (2014). Effectiveness of artesunate–amodiaquine vs. artemether–lumefantrine for the treatment of uncomplicated falciparum malaria in Nanoro, Burkina Faso: a non-inferiority randomised trial. Trop Med Int Health.

[11] Sondo P, Derra K, Diallo-Nakanabo S, Tarnagda Z, Zampa O, Kazienga A (2015). Effectiveness and safety of artemether–lumefantrine versus artesunate–amodiaquine for unsupervised treatment of uncomplicated falciparum malaria in patients of all age groups in Nanoro, Burkina Faso: a randomized open label trial. Malar J.

[12] Sondo P, Derra K, Nakanabo SD, Tarnagda Z, Kazienga A, Valea I (2017). Comparison of effectiveness of two different artemisinin-based combination therapies in an area with high seasonal transmission of malaria in Burkina Faso. Ann Parasitol.

[13] Sondo P, Derra K, Diallo Nakanabo S, Tarnagda Z, Kazienga A, Zampa O (2016). Artesunate-amodiaquine and artemether-lumefantrine therapies and selection of *Pfcrt* and *Pfmdr1* alleles in Nanoro, Burkina Faso. PLoS ONE.

[14] Lingani M, Bonkian LN, Yerbanga I, Kazienga A, Valéa I, Sorgho H (2020). In vivo/ex vivo efficacy of artemether-lumefantrine and artesunate-amodiaquine as first-line treatment for uncomplicated falciparum malaria in children: an open label randomized controlled trial in Burkina Faso. Malar J.

[15] Pekyi D, Ampromfi AA, Tinto H, Traoré-Coulibaly M, Tahita MC, PREGACT Study Group (2016). Four artemisinin-based treatments in African pregnant women with malaria. N Engl J Med.

[16] Sirima SB, Ogutu B, Lusingu JPA, Mtoro A, Mrango Z, Ouedraogo A (2016). Comparison of artesunate-mefloquine and artemether-lumefantrine fixed-dose combinations for treatment of uncomplicated *Plasmodium falciparum* malaria in children younger than 5 years in sub-Saharan Africa: a randomised, multicentre, phase 4 trial. Lancet Infect Dis.

[17] Gargano N, Madrid L, Valentini G, D’Alessandro U, Halidou T, Sirima S (2017). Efficacy and tolerability outcomes of a phase II, randomized, open-label, multicenter study of a new water-dispersible pediatric formulation of dihydroartemisinin-piperaquine for the treatment of uncomplicated *Plasmodium falciparum* malaria in African infants. Antimicrob Agents Chemother.

[18] West African Network for Clinical Trials of Antimalarial Drugs (WANECAM) (2018). Pyronaridine-artesunate or dihydroartemisinin-piperaquine versus current first-line therapies for repeated treatment of uncomplicated malaria: a randomised, multicentre, open-label, longitudinal, controlled, phase 3b/4 trial. Lancet.

[19] WHO. Assessment and monitoring of antimalarial drug efficacy for the treatment of uncomplicated falciparum malaria. Geneva, World Health Organization; 2008.

[20] Kamaliddin C, Joste V, Hubert V, Kendjo E, Argy N, Houze SJ (2019). Evaluation of PCR to monitor *Plasmodium falciparum* treatment efficacy in a nonendemicity setting. Clin Microbiol.

[21] WHO, MMV. Methods and techniques for clinical trials on antimalarial drug efficacy: Genotyping to identify parasite populations. Geneva, World Health Organization. 2008. https://www.who.int/malaria/publications/atoz/9789241596%20305/en/

[22] Felger I, Snounou G, Hastings I, Moehrle JJ, Beck HP (2020). PCR correction strategies for malaria drug trials: updates and clarifications. Lancet Infect Dis.

